# Controllable Martensite Transformation and Strain-Controlled Fatigue Behavior of a Gradient Nanostructured Austenite Stainless Steel

**DOI:** 10.3390/nano11081870

**Published:** 2021-07-21

**Authors:** Yunbo Lei, Jiuling Xu, Zhenbo Wang

**Affiliations:** Shenyang National Laboratory for Materials Science, Institute of Metal Research, Chinese Academy of Sciences, 72 Wenhua Road, Shenyang 110016, China; yblei@imr.ac.cn (Y.L.); xujiu0@gmail.com (J.X.)

**Keywords:** gradient nanostructured, surface mechanical rolling treatment, 316L stainless steel, martensite transformation, strain-controlled fatigue

## Abstract

Gradient nanostructured (GNS) surface layer with a controllable martensite fraction has been synthesized on 316L austenitic stainless steel by means of surface mechanical rolling treatment (SMRT) with temperature being controlled. The mean grain size is in the nanometer scale in the near-surface layer and increases gradually with depth. In addition, the volume fraction of martensite decreases from ~85% to 0 in the near-surface layer while the SMRT temperature increases from room temperature to 175 °C. Fatigue experiments showed that the strain-controlled fatigue properties of the GNS samples are significantly enhanced at total strain amplitudes ≥0.5%, especially in those with a dual-phase surface layer of austenite and pre-formed martensite. Analyses on fatigue mechanisms illustrated that the GNS surface layer enhances the strength-ductility synergy and suppresses the formation of surface fatigue defects during fatigue. In addition, the dual-phase structure promotes the formation of martensite and stacking faults, further enhancing fatigue properties at high strain amplitudes.

## 1. Introduction

As an important structural material with superior corrosion resistance and deformability, 316L stainless steel is widely used in industries such as nuclear power and petrochemistry [1]. However, due to its relatively low strength, fatigue resistance of 316L stainless steel is not satisfying in some circumstances when remarkable cyclic stresses are exerted. Some severe plastic deformation (SPD) approaches such as equal channel angular pressing (ECAP) and dynamic plastic deformation were applied to enhance the tensile and fatigue strengths of 316L stainless steel [2,3,4,5]. Nevertheless, it was typically found that the fatigue properties of SPD materials were significantly decreased under strain-controlled mode or at high stress amplitudes in low-cycle fatigue (LCF) regime, mostly due to their significantly decreased ductility [3,6].

Gradient nanostructured (GNS) materials, in which grain sizes increase gradually from the nanometer scale at surface to the micrometer scale in interior, were widely investigated for their outstanding performance such as high strength-ductility synergy, diffusion rate and wear resistance in recent years [7,8,9,10,11]. Significantly enhanced fatigue properties have also been obtained in different GNS materials including 316L stainless steel [12,13,14,15,16]. For example, in comparison with the coarse-grained (CG) counterpart samples, it was found that the fatigue limit (i.e., the fatigue strength at a life > 2 × 10^6^ cycles) increased ~130%, while the fatigue strength at a life ~1 × 10^3^ cycles (i.e., in LCF regime) increased ~50%, in GNS 316L samples [13]. In addition, significantly enhanced strain-controlled fatigue properties were detected in the GNS 316L stainless steel, with an increase of more than 30 times than the CG sample in fatigue life at a total strain amplitude of 0.30% [14]. However, it was noticed that the enhancement in strain-controlled fatigue properties was only achieved at total strain amplitudes less than 0.50%. At higher strain amplitudes, a comparable fatigue life was observed in the GNS and the CG 316L samples.

In dual- or multi-phase materials, the strain/stress distribution between different phases, and the crack nucleation and propagation rates along phase boundaries play important roles in their fatigue behavior. For example, enhanced strain-controlled fatigue properties were obtained in dual-phase steels containing martensite and ferrite [17,18]. By forming nanolaminated ferrite-cementite or martensite-austenite structure in a multi-phase steel, the fatigue strength was further enhanced in the LCF regime, due to the decelerated crack opening and growth process during fatigue [19]. Therefore, the introduction of dual- or multi-phase structure into the GNS sample might be an alternative solution to further enhance the fatigue properties of 316L stainless steel.

Since the face-centered-cubic austenite phases are metastable in austenitic stainless steels such as 301, 304, and 316L, they are prone to transform into body-centered-cubic martensite phases under deformation, i.e., deformation-induced martensite transformation (DIMT) [20,21,22]. For example, about 55 and 25 vol.% of austenite transformed into martensite in 304L and 316L stainless steels, respectively, after a cold-rolling reduction of 90% [21]. By means of surface mechanical rolling treatment (SMRT), a GNS surface layer containing ~80 vol.% of martensite in the near-surface layer was formed on 316L samples at room temperature [13]. Meanwhile, the DIMT is a temperature-depended process, and the volume fraction of the martensite can be changed at different deformation temperatures. By elevating the processing temperature to 280 °C, a full austenite GNS surface layer was formed on 316L stainless steel by SMRT [14].

In this work, the effects of processing temperature on the formation of GNS surface layer were studied in 316L stainless steel, so that a method to produce GNS samples with an austenite-martensite dual-phase surface layer was developed. Subsequently, strain-controlled fatigue behavior of the GNS samples was studied at strain amplitudes ≥ 0.5%.

## 2. Materials and Methods

Commercial AISI 316L stainless steel was studied in this work. Its chemical composition (in wt.%) was 0.01C, 16.21Cr, 10.20Ni, 2.12Mo, 1.10Mn, 0.41Si, 0.01S, 0.04P and balance Fe. A full austenite CG microstructure with a mean grain size of ~70 μm was obtained in the initial material after solution-annealing at 1100 °C for 60 min and subsequent water-quenching.

**Figure 1:**
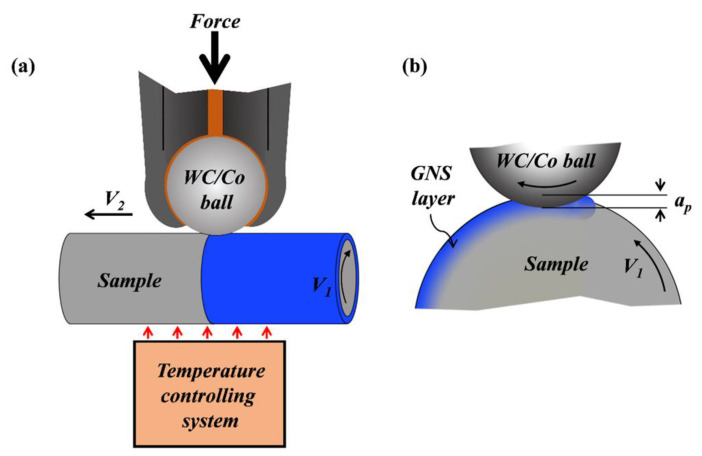
Schematic illustrations of (**a**) the SMRT set-up with a temperature controlling system and (**b**) the plastic deformation in the sample surface layer underneath the rotating ball.

Cylindrical samples were submitted to the formation of GNS surface layer at different temperatures using a SMRT system with a temperature controlling unit, in which the temperature was controlled by air heating. As schematically illustrated in Figure 1, the treated sample was heated at a preset temperature for 5 min while it rotated around the axis at a velocity of *V*_1_. Subsequently, a smooth and rotatable WC/Co ball of 8 mm in diameter was pressed into the sample surface with a penetration depth (*a_p_*), and then slid along the rod axis from one side to the other side at a velocity of *V*_2_. In this work, the SMRT process was repeated several times with the same *V*_1_ (600 rpm), *V*_2_ (0.15 mm s^−1^), and a stepwise increased *a_p_* (40 μm per step), so that a GNS surface layer might be achieved. Typically, the treated surface was smooth and containment-free, with a surface roughness (*R_a_*) of ~0.20 μm.

Microstructures of the SMRT samples were observed using transmission electron microscopy (TEM) on an FEI Talos F200X (FEI Inc, Hillsboro, OR, USA) instrument operated at 200 kV. Foils for TEM observations were prepared from cross-sectional samples containing the SMRT surface layer by electro-spark discharge machining, mechanical thinning and electro-polishing. Meanwhile, phase-constitutions in the SMRT surface layers were analyzed using X-ray diffraction (XRD) on a Rigaku D/max2400 X-ray diffractometer (12 kW) with Cu *K_α_* radiation. A step size of 2*θ* = 0.02° with counting time of 0.6 s per step was used to measure intensities of diffraction peaks in the step-scanning mode. The volume fraction of martensite (*V_α′_*) was determined according to [13,23]

(1) $$V_{\alpha^{\prime }}=\frac{1/n\sum_{j=1}^{n}I_{\alpha^{\prime }}^{j}/R_{\alpha^{\prime }}^{j}}{1/n\sum_{j=1}^{n}I_{\gamma }^{j}/R_{\gamma }^{j}+1/n\sum_{j=1}^{n}I_{\alpha^{\prime }}^{j}/R_{\alpha^{\prime }}^{j}}$$

where *n* was the number of peaks of the phase used in calculation, *I* the integrated intensity for reflecting plane, *R* the material scattering factor. The (200), (211) and (220) reflections were used for martensite, and the (200), (220) and (311) reflections were used for austenite.

Microhardness was measured using a Qness Q10 A+ (QATM, Golling, Austria) microhardness tester fitted with a Vickers indenter, with a maximum load of 20 g and a dwell time of 10 s. The measurements were performed both on the treated surface from a planar view and at different depths on the surface layer from a cross-sectional view for SMRT samples.

Cylindrical specimens with a gauge section of 6 mm in diameter were prepared for fatigue and tensile properties tests by SMRT at different temperatures. Axial tension-compression fatigue tests were performed using an Instron 8801 (Instron Inc, Massachusetts, USA) servo-hydraulic testing machine at ambient temperature. During fatigue tests, the total strain amplitude was controlled, with a strain ratio of −1, triangular wave, and a cyclic strain rate of 3.6 × 10^−2^ s^−1^. The strain amplitude was measured and controlled by a dynamic strain gauge extensometer with an accuracy higher than 10^−4^. Tensile properties were measured using an Instron 5982 (Instron Inc, Massachusetts, USA) servo-hydraulic testing machine with a strain rate of 1.2 × 10^−3^ s^−1^ at ambient temperature.

## 3. Results and Discussion

### 3.1. Formation of GNS Surface Layer with Controllable Martensite Fraction

**Figure 2:**
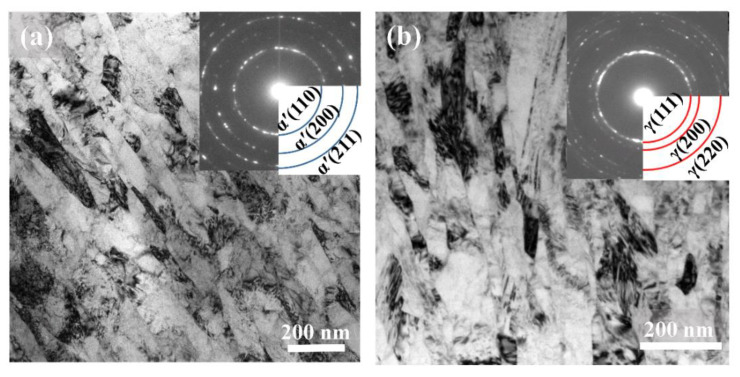
Typical bright-field TEM images at a depth of ~10 μm from the treated surface in (**a**) the SMRT-25 and (**b**) the SMRT-280 samples. Inserts show the corresponding SAED patterns.

The microstructures of the SMRT samples treated at room temperature (SMRT-25) and at 280 °C (SMRT-280) were observed by TEM. As shown in Figure 2a, elongated grains with a mean size ~45 nm along short axis and ~100 nm along long axis are formed in the near-surface layer at a depth of 10 μm in the SMRT-25 sample. From the corresponding selected area electron diffraction (SAED) pattern, it is demonstrated that the grains are mostly martensite with random crystallographic orientations. In comparison, no martensite is observed while nano-sized grains are still formed in the near-surface layer of the SMRT-280 sample. As shown in Figure 2b, austenitic grains (confirmed by the corresponding SAED pattern) with a mean size ~45 nm along short axis and ~175 nm along long axis are formed at the depth of 10 μm.

**Figure 3:**
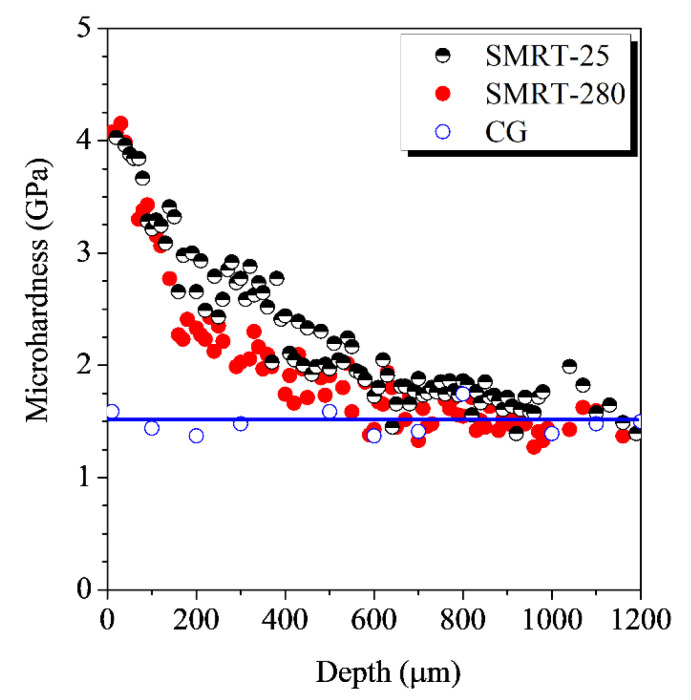
In-depth microhardness distributions in the SMRT-25 and the SMRT-280 samples, in comparison with that in the CG sample.

Further observations of microstructures at different depths demonstrated that GNS surface layers have been formed in the SMRT-25 and SMRT-280 samples, of which details can be found in the Refs. [13,14]. In the SMRT-25 sample, a mixed microstructure refinement process including dislocation activities, twinning and DIMT contributes to the formation of the GNS surface layer [13]. The mean grain size increases and the volume fraction of martensite decreases with increasing depth gradually. In comparison, no DIMT occurs during the formation of GNS surface layer and the microstructure refinement is dominated by the interaction of dislocation structures with deformation twins and lamellar structures in the SMRT-280 sample [14]. Meanwhile, gradient distributions of microhardness along depth are achieved in both the SMRT-25 and the SMRT-280 samples. As shown in Figure 3, the microhardness increases gradually from the initial value (~1.5 GPa) in the sample interior to ~4.1 GPa at the depth of ~20 μm in the SMRT-25 and SMRT-280 samples. In addition, the thicknesses of the hardened surface layers are ~800 μm in both samples. Since hardness enhancement is mostly related with the grain size refinement [24], one can deduce that GNS surface layers with a similar grain size distribution and thickness are achieved in both samples, no matter they are prepared at different temperatures.

**Figure 4:**
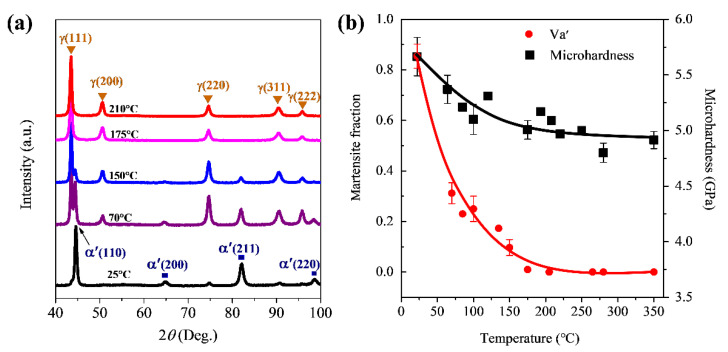
(**a**) XRD patterns measured on SMRT samples prepared at different temperatures as indicated. (**b**) Variations of martensite volume fraction and microhardness with temperature in the near-surface layer of SMRT samples.

The temperature-dependence of martensite transformation was checked by XRD measurements on the treated surface of SMRT samples. As illustrated in Figure 4a, diffraction peaks of martensite decrease and those of austenite increase in intensities with increasing temperature gradually. In addition, only austenite is detected on the SMRT samples treated at temperatures above 175 °C. According to Equation (1), the volume fraction of martensite on the treated surface reaches ~85% at room temperature and decreases gradually to almost 0 at 175 °C, as summarized in Figure 4b. Meanwhile, the surface microhardness is significantly enhanced on the SMRT samples treated at different temperatures from room temperature to 350 °C with respect to the CG sample, indicating the remarkable grain size refinement and the formation of GNS surface layer. It is noted that surface microhardness values were measured on the sample surface from a planar view in Figure 4b and on the surface layer from a cross-sectional view in Figure 3, so that difference exists in them for samples treated at the same temperature (i.e., room temperature and 280 °C). The first point measured on a cross-sectional sample should be >10 μm from the treated surface. Otherwise, the measured value may be inaccurate due to the interference between the plastic deformation zone around the indent and the sample edge.

Martensite transformation is prone to occur in austenitic stainless steel under deformation. Generally, DIMT is influenced by such factors as alloy composition, strain, strain rate, temperature, stress state, and so on. In addition, the kinetics of DIMT can be empirically modelled as [20,25]

(2) $$V_{\alpha^{\prime }}=1-\exp\left\{-\beta \lfloor 1-\exp{\left(-\alpha \varepsilon \right)}^{n}\rfloor \right\},$$

where *α* is a constant depending on stacking fault energy and strain rate, *β* is a constant relating to the chemical driving force for the martensite transformation (Δ*G*^γ^^→α^^′^), *ε* is the strain and *n* is a fixed exponent (depending on composition). Since both the stacking fault energy and Δ*G*^γ^^→α^^′^ are temperature-dependent, *α* and *β* are sensitive to temperature, i.e., decreasing to 0 with elevating temperature [20,25]. Therefore, the rate of DIMT is expected to be reduced upon deformation at higher temperatures. In addition, the volume fraction of martensite can be controlled with the sample preparation parameters (including temperature) being controlled.

Conventionally, DIMT is difficult to occur in a large scale in 316L stainless steel upon plastic strain at room temperature, due to the relatively high stability of its austenite phase. For example, only ~25 vol.% of martensite was obtained in 316L samples rolled 90% at room temperature [21]. In comparison, more than 85 vol.% of austenite in the surface layer of the SMRT-25 sample has transformed into martensite in the present work. This should be related with the fact that a high shear strain with a high strain rate is exerted in the sample surface layer by SMRT [13], which also contributes to the formation of a GNS surface layer as thick as ~800 μm even at a high temperature such as 280 °C.

### 3.2. Strain-Controlled Fatigue Properties

In a previous work [14], simultaneously enhanced stress- and strain-controlled fatigue properties were revealed in the full austenite GNS 316L stainless steel (i.e., SMRT-280). The fatigue limit increases from ~180 MPa in the CG sample to ~320 MPa in the SMRT-280 sample under stress-controlled mode. Meanwhile, the fatigue life is significantly enhanced in the SMRT-280 sample at a total strain amplitude <0.50%. However, it is found that the fatigue lives in the SMRT-280 and CG samples become comparable at higher strain amplitudes. In this work, fatigue properties of dual-phase GNS 316L samples, i.e., the SMRT-25 and SMRT-100 with austenite and martensite in the surface layer, are studied in comparison with the full austenite GNS and CG samples under strain-controlled mode at total strain amplitudes ≥0.5%.

**Figure 5:**
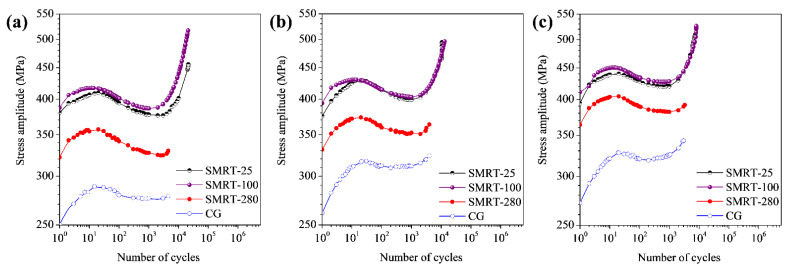
Cyclic stress amplitude curves of SMRT samples prepared at different temperatures and the CG sample during fatigue at a total strain amplitude of (**a**) 0.50%, (**b**) 0.55% and (**c**) 0.60%.

Typical cyclic stress amplitude curves were obtained from the measured stress-strain hysteresis loops with increasing fatigue cycles in different samples. As shown in Figure 5, all samples experience a rapid cyclic hardening process at the beginning of fatigue tests and a subsequent cyclic softening process. In comparison, it is found that much higher fatigue strengths are obtained in the GNS samples than in the CG samples at both stages under a same strain amplitude, while the fatigue strengths of the SMRT-25 and SMRT-100 samples are higher than that of the SMRT-280 sample. For example, the stress amplitudes of the SMRT-25, 100, 280 and CG samples are about 375, 385, 325 and 275 MPa, respectively, at the end of cyclic softening stage (i.e., the minimum values) at the strain amplitude of 0.50%.

At the final stage before fracture, a secondary hardening process (SHP) was observed. In addition, the SHP is more distinct in the SMRT-25 and SMRT-100 samples than in the SMRT-280 and CG samples. For example, the increments of stress amplitudes are typically about 80, 130, 6 and 4 MPa in the SMRT-25, 100, 280, and CG samples, respectively, after the SHP process at the strain amplitude of 0.50% (see Figure 5a). Meanwhile, it is noticed that a significantly extended fatigue endurance accompanies the SHP process in the dual-phase GNS samples, i.e., the SMRT-25 and SMRT-100 samples.

**Figure 6:**
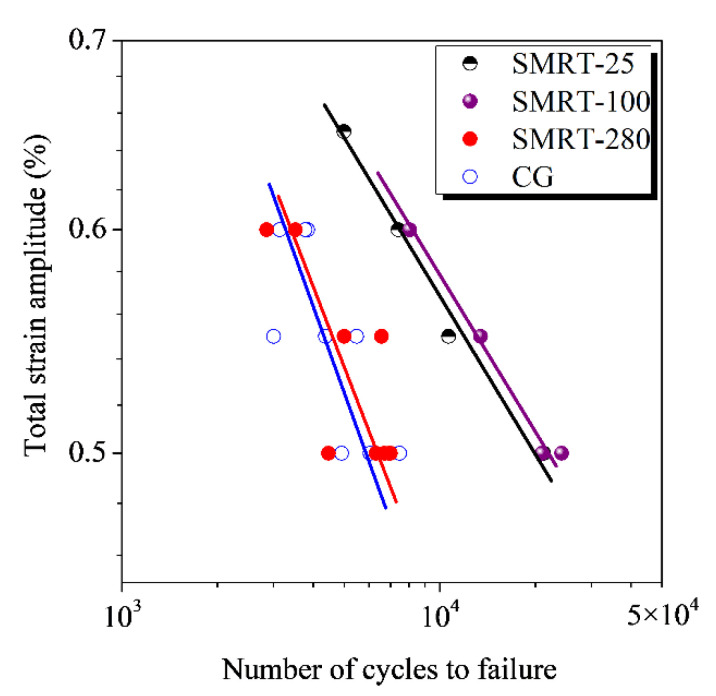
Dependences of fatigue life on total strain amplitude of SMRT samples prepared at different temperatures, in comparison with that of the CG sample.

The strain-controlled fatigue properties of the GNS and CG samples at total strain amplitudes within 0.50% to 0.60% are summarized in a plot of strain amplitude versus fatigue life in Figure 6. While comparable fatigue lives are achieved in the SMRT-280 and CG samples in a full austenite state, they are significantly enhanced in the SMRT-25 and SMRT-100 samples with a dual-phase surface layer. For example, the fatigue lives of the SMRT-25, 100, 280 and CG samples are about 2.1 × 10^4^, 2.3 × 10^4^, 6.4 × 10^3^ and 6.3 × 10^3^ cycles, respectively, at the total strain amplitude of 0.50% (or the plastic strain amplitude of ~0.25%). It is noted that the fatigue lives of the dual-phase GNS samples at a higher strain amplitude are even much higher than those of ECAP 316L samples at a lower strain amplitude, of which the fatigue lives are ~5.0 × 10^3^ (after 1-pass-ECAP) and ~2.5 × 10^3^ (after 3-pass-ECAP) cycles at the plastic strain amplitude of 0.10% [3].

### 3.3. Fatigue Mechanism

**Figure 7:**
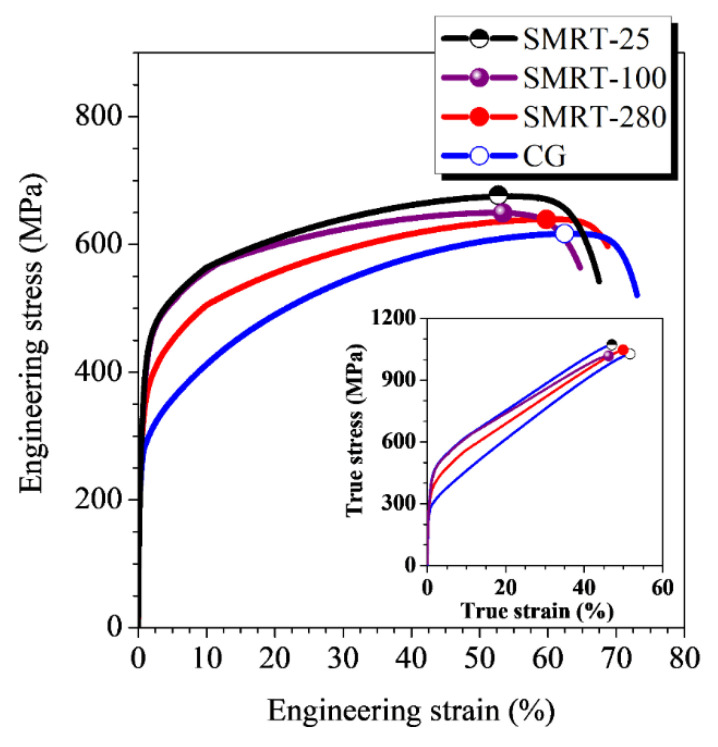
Tensile stress-strain curves of different SMRT samples and the CG sample. Insert shows the true stress-strain curves.

Significantly enhanced strain-controlled fatigue properties in the SMRT samples are primarily related with the higher strength-ductility synergy due to the formation of GNS surface layer. As revealed in previous works [7,26,27], the GNS surface layer not only enhanced the strength but also accommodated remarkable plastic deformation by suppressing strain localization during deformation of a material. This has also been confirmed by tensile tests on different SMRT samples in the present work. As demonstrated in Figure 7, the yield strengths of SMRT samples are significantly enhanced in comparison with that of the CG sample (it is noted that the true stresses of them are relatively close before necking), while the uniform and failure elongation values are still rather high (>50%). Therefore, a higher fatigue strength with considerable fatigue life is resulted in SMRT samples with respect to in CG samples fatigued at a same strain amplitude. In comparison, significantly decreased fatigue lives were obtained in nanostructured metals prepared by ECAP under strain-controlled mode, because of the significantly decreased ductility [3,6,28].

Meanwhile, the GNS surface layer contributes to the enhanced fatigue properties by suppressing the initiation of fatigue cracks on sample surface. Typically, numerous fatigue markings develop on the surface of a non-gradient material at the early stage of fatigue due to localized cyclic strains in the surface layer. For example, surface extrusions and intrusions formed on the fatigued CG 316L due to the formation of persistent slip bands [29], and surface reliefs formed on the fatigued ECAP 316L due to the dislocation slip along twin boundaries [3]. These surface defects will lead into the nucleation and propagation of cracks because of the higher stress-concentration around them, and finally result in the fatigue failure. In GNS samples, localized strains are expected to be suppressed by grain-boundary migration, confined dislocation activities between twin boundaries, improved structural homogeneity, etc. in the surface layer during fatigue [15,30,31,32], so that the developments of surface defects and cracks will be retarded. This has been confirmed by the fact that a smooth surface was kept for much longer duration on the GNS samples than on the CG counterparts under fatigue or reciprocating wear [9,13], although direct observations of the sample surfaces at different fatigue stages might provide more convincing evidence. In this case, the fatigue properties are expected to be further enhanced in addition to the contribution from enhanced strength-ductility synergy. For example, the fatigue ratio, defined as the ratio of fatigue strength to the ultimate strength, is typically higher in a GNS sample than in a non-gradient counterpart at a comparable fatigue life [13,15].

**Figure 8:**
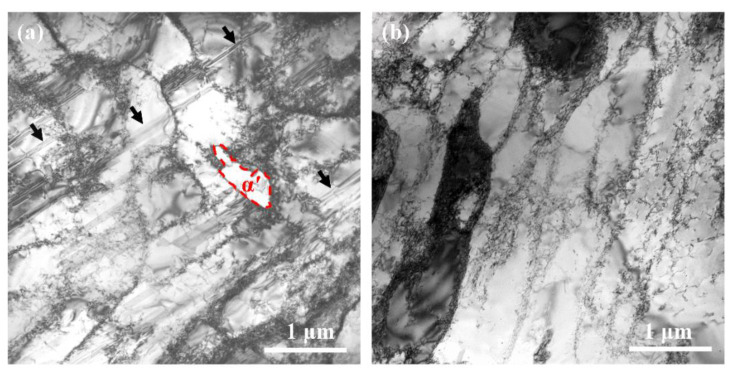
Typical TEM morphologies in the sample interior of (**a**) the SMRT-100 and (**b**) the SMRT-280 samples fatigued at a strain amplitude of 0.50%. α′-martensite (as labeled) and stacking faults (as pointed by arrows) were observed in (**a**).

In the present work, it is interesting to notice that enhanced strain-controlled fatigue properties are achieved in the GNS 316L samples with pre-formed martensite than in the full austenite GNS samples at strain amplitudes higher than 0.5%. This should be resulted from the higher accumulated deformation capacity and strain hardening ability in the dual-phase microstructure of the GNS samples under cyclic deformation. Enhanced strain-controlled fatigue properties were observed in dual-phase steels containing martensite and ferrite previously [17,18]. In addition, a higher fatigue strength and a lower crack propagation rate were also observed in a multiphase nanolaminate steel containing martensite and austenite [19]. The heterogeneous deformation, formation of dislocation substructures and cracking propensity near the phase interfaces were discussed to control the fatigue behavior of dual-phase steels. Meanwhile, the higher strain level by the accumulated strain might induce the martensite transformation and stacking faults formation in the dual-phase steel containing metastable austenite, so that the fatigue strength and life were in turn enhanced by introducing higher compressive residual stresses and deflecting crack propagation during fatigue [13,14,19,33]. In this case, further enhanced fatigue strength and extended life are expected in the GNS samples with a dual-phase (i.e., austenite and martensite) surface layer, accompanied by a distinct SHP (see the cyclic stress curves of SMRT-25 and SMRT-100 in Figure 5). As demonstrated in Figure 8, remarkable martensite grains and stacking faults were formed in the sample interior, where only austenite existed initially, in the SMRT-100 sample fatigued at a strain amplitude of 0.50%. In comparison, only dislocation structures were formed in the sample interior of the SMRT-280 sample fatigued at the same strain amplitude. Conventionally, austenite of 316L stainless steel is rather stable under cyclic deformation at room temperature. For example, almost no martensite or stacking faults was observed in the CG samples fatigued at a total strain amplitude within 0.50% to 0.60% in this work, or even up to 3.2% in Ref. [33]. In the present work, the formation of martensite and stacking faults agrees with the fact that significantly enhanced fatigue properties, i.e., a much higher strength and accumulated strain, are achieved in the dual-phase GNS samples.

## 4. Summary

GNS surface layers with different martensite fractions were formed on 316L austenitic stainless steel by means of SMRT with temperature being controlled. The volume fraction of martensite decreases gradually from ~85% at room temperature to almost 0 at 175 °C in the near-surface layer, while the thickness of GNS surface layer is similar (~800 μm) in SMRT samples prepared at different temperatures.

In comparison with CG samples, strain-controlled fatigue properties were significantly enhanced in GNS samples at total strain amplitudes ≥0.50%, especially in those with pre-formed martensite (SMRT-25 and SMRT-100). The fatigue lives of the GNS samples with pre-formed martensite are ~3 times longer, while the stress amplitudes are ~100 MPa higher, than those of the CG samples at a total strain amplitude of 0.50%. In addition to the contributions of enhanced strength-ductility synergy and suppressed surface crack initiation, the dual-phase structure (i.e., austenite and martensite) in the surface layer further enhances the accumulated deformation capacity and strain hardening ability, resulting in the significantly enhanced fatigue properties of the GNS samples at high strain amplitudes.

This work developed a simple temperature-controlled surface deformation approach to significantly enhance the strain-controlled fatigue properties of austenitic stainless steels, by which a GNS surface layer with austenite and martensite dual-phase structure is expected to form.

## Data Availability

The data presented in this study are available on reasonable requests from the corresponding author.
